# Evaluating interprofessional competency outcomes through integration of team-based interprofessional placements into an interprofessional education curriculum: a cross-sectional study

**DOI:** 10.1186/s12909-025-08256-7

**Published:** 2025-12-08

**Authors:** Takanori Uehara, Ikuko Sakai, Izumi Usui, Yoshiyuki Ohira, Masato Okubo, Itsuko Ishii, Mayumi Asahina

**Affiliations:** 1 Department of General Medicine, Chiba University Hospital, 1-8-1 Inohana, Chuo-Ku, Chiba-shi, Chiba, 260-8677 Japan; 2 Interprofessional Education Research Center, Chiba University, 1-8-1 Inohana, Chuo-Ku, Chiba-shi, Chiba, 260-8677 Japan; 3 Health Professional Development Center, Chiba University Hospital, 1-8-1 Inohana, Chuo-Ku, Chiba-shi, Chiba, 260-8677 Japan; 4 Department of General Internal Medicine, St. Marianna University School of Medicine, 2-16-1, Sugao, Miyamae-ku, Kawasaki City, Kanagawa 216-8511 Japan; 5 Division of Pharmacy, Chiba University Hospital, 1-8-1, Inohana, Chuo-Ku, Chiba-shi, Chiba, 260-8677 Japan; 6 School of Medicine, Faculty of Medicine, Toho University, 5-21-16, Omori-Nishi, Ota-ku Tokyo, 143-8540 Japan

**Keywords:** Interprofessional education (IPE), Team-based interprofessional practice placements (TIPP), Chiba interprofessional competency scale (CICS29), Team-based learning

## Abstract

**Background:**

This study aimed to evaluate whether participation in an elective, three-day Team-based interprofessional practice placement (TIPP) programme enhances interprofessional (IP) competency among medical, nursing, and pharmacy students in Japan, and to use the findings for curriculum evaluation. All students had completed a required basic interprofessional education (IPE) programme prior to participating in the TIPP.

**Methods:**

A cross-sectional survey was conducted among students enrolled between 2015 and 2018. The TIPP programme was implemented as an elective component, with recruitment methods differing by school due to differences in each school’s curriculum: nursing students who selected the TIPP programme as part of their final clinical practice were included; while medical and pharmacy students were recruited during clinical clerkships. Interprofessional competency was measured using the Chiba Interprofessional Competency Scale (CICS29), a validated self-report measure, at the time of graduation. Two-way ANOVA was performed with TIPP attendance as the main factor of interest. Bonferroni correction was applied for multiple comparisons.

**Results:**

Of the 965 students, 695 (72%) completed the CICS29, of whom 87 (13%) attended the TIPP. There was no significant interaction between TIPP attendance and school (*p* = 0.15) and no significant main effect of TIPP attendance overall (*p* = 0.12). However, post hoc comparisons showed that nursing students who attended TIPP had significantly higher total scores compared with those who did not (4.18 vs. 3.93).

**Conclusions:**

No significant main effect of TIPP participation was observed in the overall analysis, but post hoc analysis identified significantly higher CICS29 scores in nursing students, suggesting potential educational benefits. Given the short duration of the current TIPP programme and differences in the interval between TIPP participation and CICS29 measurement, future revisions should focus on extending the programme and ensuring consistent opportunities for all students to engage in authentic interprofessional learning.

**Supplementary Information:**

The online version contains supplementary material available at 10.1186/s12909-025-08256-7.

## Background

### Interprofessional education and study purpose

In 2010, the World Health Organization (WHO) highlighted the global health workforce crisis and identified interprofessional (IP) collaboration as a key solution [1]. Interprofessional education (IPE), defined by Centre for the Advancement of Interprofessional Education (CAIPE) as “occasions when two or more professions learn with, from and about each other to improve collaboration and the quality of care” (2002), was introduced in Japan in the late 20th century[2], and in recent years, increasing emphasis has been placed on clinical IPE programmes such as Team-based Interprofessional Practice Placements (TIPP). TIPP refers to a structured opportunity in which students from various health and social care professions learn together in the same setting, engaging in their typical professional roles as a collaborative, client-centred team [3]. However, implementing IPE in Japan faces challenges due to structural differences in educational curricula—medical and pharmacy curricula are six years in length, whereas the nursing curriculum is four years. In addition, preparing health professionals to work safely and effectively in dynamic clinical settings requires careful planning, intentional implementation, and evaluation [4]. 

Learner assessment plays an important role in curriculum evaluation, as it enables judgment of educational effectiveness and supports the assessment of learners’ achievements. To ensure the success of IPE programmes, evaluation frameworks must be established early and be directly aligned with longitudinal learning outcomes [5]. 

The Inohana IPE curriculum at Chiba University follows a stepwise model that progresses from foundational to applied IP learning experiences. It consists of a required four-step basic IPE programme (Steps 1–4), in which first- through fourth-year students sequentially develop IP communication, team-building, and collaborative problem-solving skills through classroom-based activities. This is followed by an advanced elective clinical programme, the TIPP programme (Step 5), which provides students with opportunities to apply these competencies in real clinical settings. Together, these five steps form an integrated, longitudinal IPE framework designed to foster the progressive development of IP collaboration competencies throughout undergraduate education.

The aim of this study was to compare the IP competencies of students—considered to be at the beginning of their professional careers as healthcare providers—based on whether or not they had participated in TIPP, and to use the results for curriculum evaluation.

In Japan, medicine and pharmacy are six-year undergraduate programmes, and nursing is a four-year undergraduate programme, all of which lead directly to national licensure examinations upon graduation. Therefore, in this study, the term “undergraduate students” refers to those enrolled in these professional degree programmes.

### Utilization of CICS29 for curriculum evaluation and educational improvement

There are best practices for curriculum development based on expert opinion, and no previous studies have reported evidence-based best practices for IPE [5]. Moreover, attempts to assess TIPP in the clinical setting are limited [6]. 

Having designed the Inohana IPE programme—named after our campus—the next challenge we faced was the objective evaluation of whether the programme was being implemented effectively and achieving its set outcomes. The Inohana IPE programme aims to cultivate autonomous healthcare professionals who demonstrate a strong commitment to patient- and client-centered care. It fosters essential competencies for IP work—including communication skills, ethical sensitivity, and problem-solving ability—while promoting continuous learning, professional responsibility, and balanced collaboration across disciplines.

To achieve this goal, we introduced the Competency in Interprofessional Collaboration Self-assessment Scale (CICS29) [7]—a validated self-assessment tool developed to measure and visualize IP competency. The CICS29 comprises six sub-domains and 29 items, rated on a five-point Likert scale, and is suitable for both educational and clinical settings. The total and six sub-domain scores are embedded in the Inohana IPE curriculum as defined learning outcomes. Incorporating CICS29 enabled systematic assessment of IP competencies in alignment with the programme’s learning goals.

## Methods

### Study design and measuring scale

This retrospective cross-sectional study used the CICS29 to evaluate the IP competency of undergraduate students at Chiba University, considering graduation as the threshold of their transition into professional healthcare roles. Although originally developed for practicing healthcare professionals, the CICS29 was adopted as a summative assessment tool for students completing their undergraduate training, in alignment with the design of the Inohana IPE programme. The CICS29 consists of 29 items across six sub-domains: (1) Attitudes and beliefs as a professional, (2) Team management skills, (3) Actions for accomplishing team goals, (4) Providing care that respects patients, (5) Attitudes and behaviors that improve team cohesion, and (6) Fulfilling one’s role as a professional. These six sub-domains reflect the key IP competencies and were used to define the Inohana IPE rubric.

These sub-domains guide the educational objectives of each step in the Inohana IPE programme and form the basis for both formative and summative assessments. The total score (maximum 145 points) indicates overall IP competency, while sub-domain scores highlight individual strengths and areas for improvement. The CICS29 has demonstrated satisfactory reliability and validity, with Cronbach’s alpha exceeding 0.80 for all sub-domains and intraclass correlation coefficients of around 0.70[8]. These qualities support its use as an outcome measure in both educational and clinical settings.

### Educational intervention: required basic IPE (step1〜4) and TIPP (step 5) programme

We embarked on the development of Chiba University’s IPE programme, “Inohana IPE”. Our IPE curriculum at Chiba University is conformed to the guidelines for reporting team-based learning implementations [9] as well as the five best practices for the development of foundational IPE programming indicated by Shrader et al.[5] In addition, Inohana IPE involves cumulative classroom IPE activities, where students experience team building using Tuckman’s model [10] in experiential workshops within the classroom IPE. This structured, cumulative educational intervention spans all academic years and is integrated into the formal undergraduate curriculum for students in the schools of medicine, nursing, and pharmacy. We gathered information on IPE in the UK since 2005 and initiated Inohana IPE Step 1 in 2007, targeting first-year students from medical, nursing, and pharmacy schools. We sequentially launched Steps 2, 3, and 4 for second-, third-, and fourth-year students, respectively, resulting in the completion of a four-step annual cumulative required basic IPE programme in 2010. Each step focuses on progressively developing IP collaborative competencies through classroom and experiential learning. For detailed descriptions of the curriculum structure, objectives, and instructional methods of the required basic Inohana IPE programme, please refer to the official programme website: https://www.n.chiba-u.jp/iperc/home-english/inohana-ipe/contentsandsystem/index.html (in English).

**Fig. 1:**
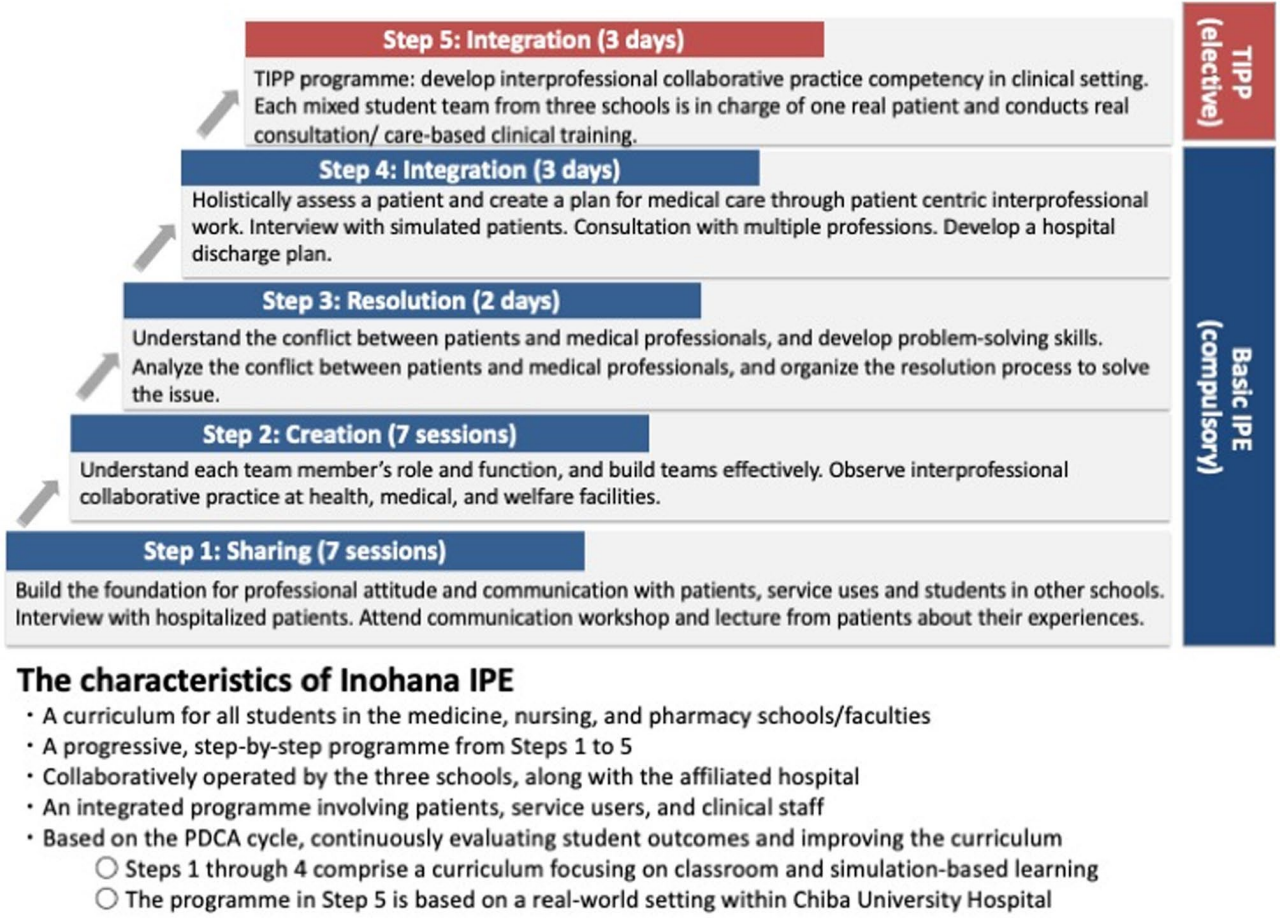
Basic IPE and TIPP programme, and the characteristics of Inohana IPE. *TIPP* Team-based interprofessional practice placement, *IPE* Interprofessional education

Following from the required basic IPE programme, the need arose for TIPP that could be applied in real-world clinical practice. In actual clinical settings, students are expected not only to build their own teams but also to integrate into existing clinical teams. TIPP, whose programme is based on real-world settings, intends to foster students’ competency in team management and performance by making assessments and plans regarding patient-user-centred problem-solving under clinical clerkship. Students collaborate with clinical professionals to manage patient-centered problems through team-based assessments and care planning during TIPP. In 2015, we implemented the TIPP programme (Step 5) as a three-day elective for fifth-year medical and pharmacy students and fourth-year nursing students, aiming to apply the foundational competencies acquired in Steps 1–4 to real-world clinical contexts. As illustrated in Fig. 1, the programme consists of a required four-step basic IPE component (Steps 1–4) delivered annually from the first through fourth year, followed by an elective advanced IPE component, the TIPP programme, as Step 5 in the final academic year.

The detailed TIPP programme is outlined below. The TIPP programme is conducted in-person in clinical wards such as the Department of Pediatrics and the Division of Diabetes, Metabolism, and Endocrinology at Chiba University Hospital. Students from the three schools form interdisciplinary teams, with each team responsible for one patient. Over the course of three days, the team collaboratively develops a care plan tailored to their assigned patient. On the first day, the team meets with the patient and participates in discipline-specific activities (e.g., observation or practice sessions) in accordance with the patient’s schedule. Daily team discussions and individual feedback sessions with clinical supervisors are held throughout the programme. On the final day, students present their care plans and reflect on the care they implemented. Each team is guided by a group of approximately five supervisors from various health professions. In addition to the CICS29, students were asked to complete supplementary questionnaires to evaluate the TIPP programme.

Furthermore, to establish the learning goals of the Inohana IPE programme, we conducted a pilot study to develop a scale measuring IP collaborative practice competency among healthcare professionals and extracted six factors from Yamamoto el al[8]: (a) Respecting Patients, (b) Team Management Skills, (c) Fulfilling One’s Role as a Professional, (d) Attitudes and Beliefs as a Professional, (e) Attitudes that Improve Team Cohesion, and (f) Taken to Achieve the Team’s Goal. Based on these six factors, we developed a rubric to define the learning goals for each step of our Inohana IPE programme. Additionally, the learning objectives of the TIPP programme are aligned with six core sub-domains of IP collaborative practice competency. By the end of the TIPP programme, students are expected to demonstrate the following abilities, as described in the TIPP rubric:

1. Actions to achieve team goals: Assess the clinical setting and the condition of the team, engage in professional practice, and provide constructive feedback to team members to achieve patient-centered goals.
2. Team management skills: Share information and make collaborative decisions with team members, taking into account the expertise and limitations of each profession.
3. Attitudes that improve team cohesion: Build good interpersonal relationships with diverse professionals in clinical settings to promote a supportive atmosphere for patient-centered care.
4. Providing care that respects patients: Implement and evaluate the care plan developed by the team, while respecting the patient’s wishes.
5. Professional attitudes and beliefs: Incorporate up-to-date knowledge from each field into the planning, implementation, and evaluation of care, with support from supervising professionals and faculty members.
6. Fulfilling one’s role as a professional: Reflect on and carry out tasks appropriate to one’s professional scope from a student perspective, and provide feedback to the clinical team.

### Participants and setting

The participants were students from the schools of medicine, nursing, and pharmacy at Chiba University, Japan. Due to differences in the non-IPE curricula across schools, both the recruitment procedures for participation in the TIPP programme and the timing between the training and the administration of the CICS29 differed. To improve the response rate, we employed different methods of questionnaire collection for each school. These adjustments were necessitated by the distinct curricular structures of the respective programmes. Nursing students who selected the TIPP programme as part of their final clinical practice were included in the study, whereas only a proportion of students in the six-year medical and pharmacy programmes who were in clinical clerkship rotations during the period when TIPP was held (between July 2015 and 2018) were invited to participate. All students, regardless of their school, completed the CICS29 only once after participating in the TIPP programme, but the timing of administration differed by school due to variations in their curricula. These students completed the CICS29 at the time of graduation; however, the timing of graduation in relation to the TIPP programme varied. Medical students completed the CICS29 once, after finishing their graduation examinations, which were computer-based tests conducted one and a half years after the TIPP programme, following one year of clinical clerkship. Pharmacy students completed the CICS29 only once, at the time of their graduation ceremonies, which were held one and a half years after the TIPP programme, following one year of experimental research. Nursing students completed the CICS29 at the time of their graduation ceremonies, which occurred six months after the TIPP programme. Our study included students who graduated between 2015 and 2018 and fully completed the CICS29 questionnaires. The CICS29 was administered immediately after the examination sessions and took approximately 10 min to complete.

### Main outcome

This study aimed to examine differences in the mean total and sub-domain scores of the CICS29 (Appendix 1) between students who participated in the TIPP programme and those who did not. CICS29 was developed and validated through a series of prior studies by Yamamoto et al.[8], in collaboration with over 2,000 healthcare professionals [7]. Given that the CICS29 is deliberately embedded in the Inohana IPE programme as a curriculum-integrated rubric, the total score serves as a summative indicator of overall IP competency, while the six sub-domain scores reflect specific learning outcomes defined within the programme. Responses to each item were recorded using a five-point Likert scale, ranging from 5 (always) to 1 (never). Subgroup analyses were also conducted within each school (medicine, nursing, and pharmacy) to explore potential differences associated with TIPP participation.

### Statistical analysis

We analysed the main outcomes using two-way ANOVA with factors for school and TIPP participation. Post hoc comparisons within each school were conducted using Bonferroni correction for multiple comparisons. All statistical analyses were performed using SPSS for Windows version 25.0 (IBM Corporation, Armonk, NY, USA), with significance set at *p* < 0.05.

### Ethical considerations

The present study was approved by the Research Ethics Committee of Chiba University School of Nursing. We provided each student with a document explaining the ethical considerations of the educational initiative. Participants completed informed consent forms before participating.

## Results

### Characteristics of participants

**Fig. 2:**
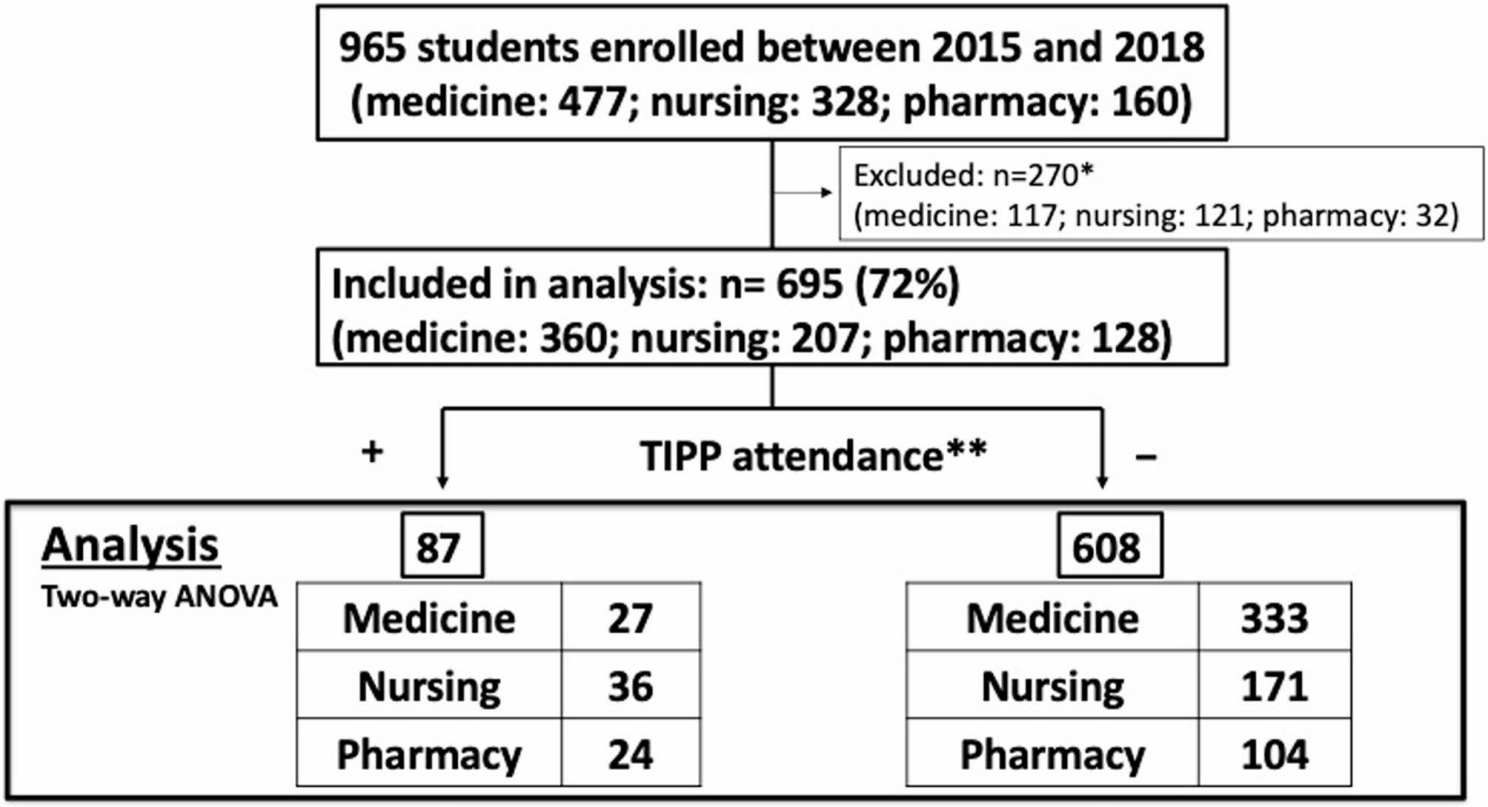
Characteristics of the participants. *TIPP* Team-based interprofessional practice placement, *CICS29* Chiba Interprofessional Competency Scale

Of the 965 eligible students (medicine: 477; nursing: 328; pharmacy: 160), 695 (72%) completed the CICS29 and were included in the analysis. The response rates by school were 75.5% for medical students (360/477), 63.1% for nursing students (207/328), and 80.0% for pharmacy students (128/160). Figure 2 presents the characteristics of the 695 participating students who completed the CICS29. Of these students, 87 (13%) participated in the TIPP programme (medicine: 27; nursing: 36; pharmacy: 24). Students who did not complete all 29 items were excluded from the analysis.

### Comparison of CICS29 total and sub-domain scores by TIPP participation

In the two-way ANOVA, no significant main effect of TIPP participation was observed for the total CICS29 score (*p* = 0.12), nor was there a significant interaction between school and TIPP participation (*p* = 0.15) (Table 1). A significant main effect of TIPP participation was found only for sub-domain 1 (Attitudes and beliefs as a professional) (*p* = 0.03).

**Table 1:** Mean CICS29 total and sub-domain scores with TIPP or non-attendance for each school (2-way ANOVA) *n* = 695

CICS29 total and sub-domain	TIPP	M mean (95% CI)	*N* mean (95% CI)	*P* mean (95% CI)	Interaction between TIPP and school	Main effect of TIPP
CICS29 total scores	- +	4.05 (4.00–4.11.00.11) 4.08 (3.88–4.27) *p* = 0.81	3.93 (3.85–4.01) 4.18 (4.02–4.35) *p* < 0.01	3.87 (3.77–3.96) 3.87 (3.67–4.08) *p* = 0.97	F(2, 689) = 1.93 *p* = 0.15	*p* = 0.12
1. Attitudes and beliefs as a professional	- +	4.03 (3.97–4.09) 4.08 (3.87–4.29) *p* =0.63	3.78 (3.70–3.87) 4.09 (3.91–4.27) *p* < 0.01	3.83 (3.72–3.93) 3.90 (3.67–4.12) *p* = 0.59	F(2, 689) = 1.71 *p* = 0.181	*p* = 0.03
2. Team management skills	- +	4.01 (3.94–4.07) 4.01 (3.79–4.22) *p* = 0.99	3.86 (3.77–3.94) 4.11 (3.92–4.29) *p* = 0.02	3.78 (3.67–3.89) 3.78 (3.56–4.01) *p* = 0.97	F(2, 689) = 1.68 *p* = 0.19	*p* = 0.20
3. Actions for accomplishing team goals	- +	4.03 (3.97–4.09) 4.09 (3.87–4.31) *p* = 0.59	3.83 (3.75–3.92) 4.06 (3.87–4.25) *p* = 0.04	3.80 (3.69–3.91) 3.87 (3.64–4.10) *p* = 0.60	F(2, 689) = 0.67 *p* = 0.51	*p* = 0.08
4. Providing care that respects patients	- +	4.23 (4.18–4.29) 4.25 (4.06–4.45) *p* = 0.84	4.28 (4.20–4.35) 4.42 (4.25–4.59) *p* = 0.12	4.13 (4.03–4.23) 3.90 (3.69–4.11) *p* = 0.12	*F(2, 689)* *= 3.13* *p = 0.04*	*p* = 0.74
5. Attitudes and behaviors that improve team cohesion	- +	4.02 (3.96–4.09) 3.95 (3.72–4.18) *p* = 0.57	3.98 (3.89–4.07) 4.29 (4.09–4.49) *p* < 0.01	3.78 (3.67–3.90) 3.87 (3.62–4.11) *p* = 0.56	F(2, 689) = 2.64 *p* = 0.07	*p* = 0.14
6. Fulfilling one’s role as a professional	- +	3.98 (3.91–4.05) 4.04 (3.80–4.27) *p* = 0.64	3.88 (3.79–3.97) 4.18 (3.98–4.38) *p* < 0.01	3.87 (3.75–3.99) 3.92 (3.67–4.16) *p* = 0.73	F(2, 689) = 1.41 *p* = 0.24	*p* = 0.06

The numbers in parentheses are the order in which the three schools are listed

*M* Medicine, *N* Nursing, *P* Pharmacy, *N.S.* Not significant, *TIPP* Team-based interprofessional practice placement, *CICS29* Chiba Interprofessional Competency Scale

Post hoc comparisons within each school using Bonferroni correction revealed that nursing students who participated in the TIPP programme scored significantly higher than non-participants in the total CICS29 score and in sub-domains 1, 2, 3, 5, and 6 (*p* < 0.05). In contrast, no significant differences were found between TIPP participants and non-participants among medical and pharmacy students in any sub-domain.

## Discussion

The two-way ANOVA showed no significant main effect of TIPP participation or interaction with school for the CICS29 total score. While the results should be interpreted with caution due to the multiple comparisons and context-specific nature of the findings, the post hoc analysis revealed a significant increase in scores among nursing students who participated in the TIPP programme. We believe that the primary reasons for the lack of a significant main effect of TIPP participation were the short duration of the TIPP programme (only three days) and the unavoidable differences in the timing of CICS29 score measurement due to variations in each school’s curriculum. A previous study reported that the duration of TIPP should be at least two weeks to be effective [11]. Our TIPP programme is designed as an elective extracurricular component that builds upon the required basic IPE curriculum. However, due to scheduling constraints within the existing curricula of the three participating schools, the programme is currently limited to a three-day period in July. This limited duration represents a significant challenge. Follow-up evaluations of former students who trained in TIPP have indicated that IPE conducted in training wards was valuable to them and that they applied the experience gained through TIPP in their later practice [12]. Curriculum coordination and integration are recognized as major issues in IPE [5], and the TIPP programme should be formally incorporating into the standard curricula of all three schools and extended from three days to approximately two weeks to enhance its educational impact. A study on the Advanced Life Support in Obstetrics (ALSO) course, which aims to support the acquisition and maintenance of knowledge and skills necessary for managing obstetric emergencies, reported a significant decline in self-assessed confidence and knowledge when comparing immediately after the course and six weeks later[13]. In the present study, the interval between TIPP participation and the CICS29 evaluation was relatively short for nursing students, whereas it was longer for medical and pharmacy students. Therefore, it is important to provide continuous training opportunities even after TIPP participation and to establish a system of post-training supervision and follow-up. Furthermore, to clarify how long the effects of TIPP are sustained, future studies should compare CICS29 scores immediately after the programme and after a certain period, in order to determine the optimal timing for TIPP delivery and the need for refresher training. In the post hoc analysis, a significant improvement in the CICS29 total score was observed among nursing students who participated in the TIPP program, whereas no such improvement was found among medical and pharmacy students. This finding suggests that, in addition to other factors, differences in participant recruitment methods may have contributed to the lack of a significant main effect of TIPP participation. In the TIPP program, nursing students included those who actively volunteered to participate, whereas medical and pharmacy students agreed to participate upon request during their clinical clerkship rotations. It has been reported that learners who self-select for IPE tend to be more motivated to engage in collaborative learning with other professional groups [12]. On the other hand, clinical IPE programmes, including TIPP, represent essential educational opportunities that serve as a foundation for lifelong learning among all healthcare professionals, including students. Therefore, it is necessary to consider the design and implementation of attractive TIPP programmes that enable all students to participate proactively—for example, through faculty development, threading curricular content aligned with real-world practice environments, utilizing pilot data to refine the programme, and actively publicising the initiative [5].

The analysis of the CICS29 sub-domains yielded results that were largely consistent with those of the total score analysis. A significant main effect of TIPP participation was not observed in sub-domains 2 through 5, except for sub-domain 1. Among nursing students, a significant difference based on TIPP participation was observed in all sub-domains except sub-domain 4. The higher scores observed in nursing students may reflect the greater emphasis placed on direct patient interaction and team-based practice within their curriculum. The CICS29 is a scale designed to obtain a multidimensional measurement of competencies in IP collaborative practice, and the Inohana IPE programme has developed its educational rubric based on the six sub-domains of the CICS29. Incorporating such sub-domain-specific insights into curriculum evaluation enables the identification of targeted areas for improvement across different healthcare disciplines. The critical point, as noted by Hoti et al.[14], is that curriculum improvement becomes possible when specific issues are identified. In line with our discussion on the CICS29 total score, the results from the sub-domain analysis should be utilized to clarify which components of IP competencies require focused instruction, and these findings should be applied to the ongoing development of the curriculum.

This study aimed to examine whether an elective TIPP programme could enhance IP competencies and to contribute to the improvement of the programme. Therefore, differences among schools were not included as a primary focus of analysis; however, we would like to briefly address this issue here. As a premise of the Inohana IPE programme, all students from the three schools are assumed to have an equivalent level of readiness for IPE, as they have completed the required basic IPE programme consisting of Steps 1 through 4 in a sequential and cumulative manner. Nevertheless, differences in CICS29 scores across disciplines were observed, which may represent an issue to be explored in future research. In particular, pharmacy students showed the lowest scores in both the total score and all sub-domains, suggesting that school-specific factors—such as the longer interval between TIPP participation and the CICS29 evaluation, as well as limited clinical exposure during the research-focused period following TIPP—may influence the outcomes of IP learning. These findings suggest that pharmacy students may require more targeted opportunities for authentic IP experiences during their curriculum, particularly given the limited clinical exposure following TIPP. Incorporating longitudinal IPE activities or embedding pharmacists more actively in team-based clinical settings may help strengthen their IP competencies. However, as seen in studies comparing students from Indonesia [15] and those from Finland and Sweden[16], measured IP competency outcomes may vary across countries due to differences in educational structures, healthcare systems, and cultural norms. This suggests that benchmarking or interpreting results must be done with careful consideration of national context, and not assumed to follow a universal pattern.

We aimed to improve the TIPP programme by evaluating differences in outcomes based on TIPP participation. While there is currently no unified scale for measuring IP competency, it is essential to use a consistent scale such as the CICS29 to allow for comparisons across cohorts and time points in future studies. Although our current study is cross-sectional, applying the same multidimensional instrument for assessing IP competency over time can support cumulative curriculum evaluation. Enhancing the team-building skills of the teams involved in TIPP as well as securing sufficient time for these teams to operate effectively present significant challenges.

### Limitations of the evaluation of each school

The evaluation of this educational initiation across the three schools at Chiba University has some limitations. First, the initiative was conducted in a single university; therefore, any generalisations should be treated with caution. Moreover, our research did not confirm whether the non-respondents’ perceptions would be consistent with those of surveyed students. Second, given the difficulty in integrating the three schools’ curricula completely, we could not unify the interval of intervention (IPE and TIPP) and outcome (examination). Third, the use of CICS29 as a self-assessment tool may involve limitations inherent to subjective measurement, such as self-perception bias and the possible gap between perceived and actual competency levels. Previous literature has suggested that perceived improvement in competence, particularly among novice learners, may not consistently align with objectively measured abilities [17]. While self-assessment provides valuable insight into learners’ own perspectives, it may not fully reflect the impact of educational interventions when compared with other levels of the Kirkpatrick model, such as observed behavior or outcome measures. Therefore, combining self-assessment with additional objective indicators— such as external assessments or observed team performance— might offer a more comprehensive understanding of IPE outcomes.

Fourth, IP competency development may be influenced not only by TIPP but also by discipline-specific training experiences such as clinical clerkships or laboratory-based learning. The extent to which these experiences incorporate IP elements varies among disciplines, and this variation could act as a confounding factor in interpreting the results.

## Conclusions

Although no significant main effect of TIPP participation was observed in the overall analysis, post hoc analysis identified that nursing students who participated in the TIPP programme had significantly higher CICS29 scores, suggesting potential educational benefits. Given the short duration of the current TIPP programme and differences in the interval between TIPP participation and CICS29 measurement, future revisions should focus on extending the programme and ensuring consistent opportunities for all students to engage in authentic IP learning.

## Supplementary Information

Supplementary Material 1.

## Data Availability

The data utilized in this study are not publicly available because the relevant quotes are included in the results section. However, they can be made available by the corresponding author upon reasonable request.

## Abbreviations

TIPP: Team-based interprofessional practice placement
CICS29: Chiba Interprofessional Competency Scale
IP: Interprofessional
IPE: Interprofessional education
