# Supplementary material for: Evaluating interprofessional competency outcomes through integration of team-based interprofessional placements into an interprofessional education curriculum: a cross-sectional study
Source: BMC Med Educ. 2025 Dec 8;25:1681. doi: 10.1186/s12909-025-08256-7 (PMC12683858; doi:10.1186/s12909-025-08256-7)
Supplement: Supplementary file 1 — Supplementary Material 1. [file 12909_2025_8256_MOESM1_ESM.docx]

Appendix 1. The CICS29: 29 items in six domains

A five-point Likert scale was used to record the responses to each item, ranging from 1 (never) to 5 (always). The reliability of the instrument is indicated by Cronbach’s alpha exceeding 0.80 for all sub-domains and intraclass correlation coefficients of around 0.70.

|  | Sub-domain | Item |
| --- | --- | --- |
| 1 | Domain 1. Attitudes and beliefs as a professional | I constantly strive to improve my performance. |
| 2 |  | I always reflect on the care that I have provided. |
| 3 |  | I strive to be a professional. |
| 4 |  | I practice evidence-based care. |
| 5 |  | I am able to explain the basis for care to anyone. |
| 6 |  | I am able to apply updated expert knowledge to actual practice. |
| 7 | Domain 2. Team management skills | I understand the scope and limits of my team members’ work. |
| 8 |  | I respect my team members’ busy schedules and work pace. |
| 9 |  | I cooperate with my team members to try to solve the problem when the team is not functioning well. |
| 10 |  | I reconcile conflicts among team members. |
| 11 |  | I know when problems within the team are likely to arise. |
| 12 | Domain 3. Actions for accomplishing team goals | I am able to explain the results of my team’s initiatives. |
| 13 |  | I am able to adjust my practice to achieve the team’s objectives. |
| 14 |  | I am able to coordinate the opinions of myself and my team members in light of the team’s objectives. |
| 15 |  | I provide the necessary support to my team members depending on their professional competency. |
| 16 |  | I am able to evaluate objectively whether the team is operating well. |
| 17 | Domain 4. Providing care that respects patients | I respect not only the wishes of the patients but also those of their families. |
| 18 |  | I keep the patients’ independence in mind when providing care. |
| 19 |  | I interact with patients to help them make their own decisions. |
| 20 |  | I change my manner of interacting with patients on the basis of their characteristics and situations. |
| 21 |  | I seek the best way to care for patients. |
| 22 | Domain 5. Attitudes and behaviors that improve team cohesion | I consciously create opportunities for communication with other professionals. |
| 23 |  | I discuss ideal patient care daily with other professionals. |
| 24 |  | I try to create a suitable atmosphere during meetings wherein it is easy for other professionals to speak. |
| 25 |  | I strive daily to create good interpersonal relations with other professionals. |
| 26 | Domain 6. Fulfilling one’s role as a professional | I am able to express opinions in front of other professionals on the basis of my expert knowledge. |
| 27 |  | I fulfill my professional role as required by my team. |
| 28 |  | I understand the scope of what can be accomplished by professional expertise and skills. |
| 29 |  | I am able to state my opinions when necessary based on my professional expertise, even if doing so creates friction with other professionals. |
